# Adenosine-Mono-Phosphate-Activated Protein Kinase-Independent Effects of Metformin in T Cells

**DOI:** 10.1371/journal.pone.0106710

**Published:** 2014-09-02

**Authors:** Marouan Zarrouk, David K. Finlay, Marc Foretz, Benoit Viollet, Doreen A. Cantrell

**Affiliations:** 1 Division of Cell Signalling and Immunology, College of Life Sciences, University of Dundee, Dundee, Scotland, United Kingdom; 2 School of Biochemistry and Immunology, Trinity Biomedical Sciences Institute, Trinity College Dublin, Dublin, Ireland; 3 School of Pharmacy and Pharmaceutical Sciences, Trinity Biomedical Sciences Institute, Trinity College Dublin, Dublin, Ireland; 4 Institut Nationale de la Santé et de la Recherche Médicale Unité 1016, Institut Cochin, Paris, France; 5 Centre Nationale de la Recherche Scientifique, Unités Mixtes de Recherche 8104, Paris, France; 6 Université Paris Descartes, Sorbonne Paris Cité, Paris, France; University of Oklahoma Health Science Center, United States of America

## Abstract

The anti-diabetic drug metformin regulates T-cell responses to immune activation and is proposed to function by regulating the energy-stress-sensing adenosine-monophosphate-activated protein kinase (AMPK). However, the molecular details of how metformin controls T cell immune responses have not been studied nor is there any direct evidence that metformin acts on T cells via AMPK. Here, we report that metformin regulates cell growth and proliferation of antigen-activated T cells by modulating the metabolic reprogramming that is required for effector T cell differentiation. Metformin thus inhibits the mammalian target of rapamycin complex I signalling pathway and prevents the expression of the transcription factors c-Myc and hypoxia-inducible factor 1 alpha. However, the inhibitory effects of metformin on T cells did not depend on the expression of AMPK in T cells. Accordingly, experiments with metformin inform about the importance of metabolic reprogramming for T cell immune responses but do not inform about the importance of AMPK.

## Introduction

T lymphocytes respond to pathogens by differentiating to effector subpopulations that mediate the protective immune response. Effector T cells strikingly increase their cellular uptake of multiple nutrients including glucose, amino acids and transferrin. They also swap from metabolising glucose primarily through oxidative phosphorylation to become highly glycolytic [1]–[4]. The changes in effector T cell metabolism are important as judged by the consequences of inhibiting key metabolic regulators. For example, the serine/threonine kinase mTORC1 (mammalian Target Of Rapamycin Complex 1) integrates inputs from nutrients, antigen and cytokine receptors to link T cell metabolism and T cell differentiation [5]. mTORC1 thus controls expression of cytolytic effector molecules, chemokine and adhesion receptors in effector T cells [3], [6] and controls effector-memory cell transition [7], [8].

One other regulator of T cell differentiation is the adenosine-monophosphate (AMP)-activated protein kinase (AMPK) [9], [10]. AMPK is phosphorylated and activated by liver kinase B1 (LKB1) in response to energy stress and functions to enforce quiescence to restore energy balance in cells [11]. In T lymphocytes, AMPK is important for the transition of effector T lymphocytes to memory T cells during the contraction phase of the immune response [10]. Hence as inflammatory signals fade during the resolution of immune responses, signalling via AMPK allows T effector cells to resume a metabolically quiescent state so that they persist to produce accelerated responses upon secondary infection [10].

The idea that AMPK is an important regulator of T cell functions has been strengthened by the observations that metformin, a drug that activates AMPK, inhibits the production of effector T lymphocytes and promotes the production of memory T cells [12]–[14]. The anti-inflammatory actions of metformin extend to its ability to suppress the development of autoimmune diseases in mouse models [12], [15]. Moreover, metformin has been shown to inhibit the proliferation and survival of acute myeloid leukaemic [16] and T-cell acute lymphoblastic leukaemic cells [17], [18].

Metformin activates AMPK because this drug inhibits respiratory chain complex I and thereby causes an increase in the cellular AMP/ATP ratio [19], leading to the phosphorylation and activation of AMPK via LKB1 [11]. The effects of metformin on T cell function are thus invariably interpreted in terms of its ability to activate AMPK. Indeed, current models of AMPK function in immune cells are based largely on experiments with metformin. There is, however, a critical caveat because metformin only indirectly activates AMPK, because it inhibits respiratory chain complex I and thereby causes an increase in cellular AMP/ATP ratio. Metformin thus has many effects on cell metabolism that are not mediated by AMPK [20]–[22]. Indeed, even the actions of metformin in the liver that underpin its efficacy in the treatment of diabetes have been shown to be AMPK-independent [20], [22].

The potential for AMPK-independent actions of metformin does not seem to be considered in any of the immunological studies that use this drug to manipulate cellular immune responses. Consequently, the regulatory effects of metformin in the immune system are used to model the role of AMPK. Accordingly, the objective of the present study is to explore the relevance of AMPK in mediating the immune-regulatory effects of metformin in T lymphocytes. We compared the effects of metformin on antigen receptor and cytokine regulated responses in wild type and AMPKα1^null^ CD8 T cells. We found that metformin controls key metabolic pathways in T cells and hence controls T cell growth and proliferation. However, the immune regulatory effects of metformin have no requirement for expression of AMPK in T cells. Experiments with metformin thus inform about the importance of metabolic signalling for T cell biology but do not inform about the role of AMPK.

## Materials and Methods

### Ethics Statement

Mice, OT-I TCR^+^ transgenic or AMPKα1^fl/fl^ CD4Cre^+^, were bred and maintained under specific pathogen-free conditions in the Biological Resource Unit at the University of Dundee. The procedures used were approved by the University Ethical Review Committee, a committee of the University Court, at its meeting on 19^th^ December 2007 and subsequently authorised by a project licence according to the UK Home Office Animals (Scientific Procedures) Act 1986 as issued by the Home Office on 14^th^ April 2008.

### Lymphocyte Culture

CD8^pos^ lymphocytes were isolated by autoMACS using the CD8α+ T cell Isolation Kit II (Miltenyi Biotec, Germany). CTL cultures from OT-I TCR^+^ mice were generated as previously described [3], [10], [23]. Where outlined, cultures were also treated with 10 mM metformin (1,1-dimethylbiguanide hydrochloride; Sigma-Aldrich). Where shown, freshly isolated lymphocyte cultures were maintained in 5 ng mL^−1^ IL-7 (Peprotech) for indicated periods of time. For proliferation assays, lymph node suspensions from OT-I TCR^+^ mice were labelled with 5 µM CFSE or Cell Tracer Violet (Molecular Probes) and TCR activated with 1 nM SIINFEKL in the absence or presence of 10 mM metformin for up to 72 h.

### Immunoblotting

Lymphocytes were lysed in F buffer (1×10^7^ cells mL^−1^) and subjected to immunoblot analysis as described previously [3], [10]. Total ACC and ACC^S79^ were detected by bi-fluorometric analysis using the Odyssey LICOR system and ImageJ software was used for integral signal quantification. Anti-AMPKα1 and anti-ACC^S79^ were kindly provided by Grahame Hardie, University of Dundee, U.K. Anti-Smc1 was purchased from Bethyl Laboratories Inc and anti-Hif1α was obtained from R&D Systems. Anti-Glut1 was a gift from Geoff Holman, University of Bath, U.K. [19], [24]. All other antibodies for immunoblotting were obtained from Cell Signaling Technology. Antigens were detected using suitable HRP-conjugated secondary antibodies and enhanced chemoluminescence.

### Flow Cytometry

Accurate cell counts of lymphocyte cultures were taken by using AccuCheck counting beads (Life Technologies, UK) or, alternatively, by direct event counts against volumetric flow rate on FACSVerse (Beckton Dickinson). Antibodies used for flow cytometry were conjugated to fluorescein-isothiocyanate (FITC), phycoerythrin (PE), peridinin-chlorophyll protein (PerCP)-Cy.5.5, PE-Cy7, allophycocyanin (APC), APC-eFluor 780 or Alexa Fluor 647 and were obtained from BD Pharmingen or eBiosciences unless otherwise stated: anti-CD8α (clone 53-6.7), anti-CD25 (clone PC61), anti-CD44 (clone IM7), anti-CD69 (clone H1.2F3), CD71 (clone C2), CD98 (clone RL388; Biolegend). Intracellular levels of S6 protein phosphorylated on S235 and S236 (S6^S235/6^) were detected by using Alexa-Fluor-647-conjugated anti-S6^S235/6^ (4851; Cell Signalling Technologies) as previously described [23]. Following incubation with antibodies, cells were washed and re-suspended in FACS buffer. Samples were analysed using LSR II Fortessa or FACSVerse (Becton Dickinson). A minimum of 1×10^4^ ungated events were acquired and stored. Data files were processed using the FlowJo software V9.6.4 (Treestar) for Mac OS. Live cells were gated according to their forward and side scatters.

### Metabolic Assays

Glucose uptake and lactate output assays were performed as previously described [3], [23].

### Statistical Analysis

Quantified data were statistically evaluated using non-parametric Mann-Whitney test or two-way ANOVA with Bonferroni’s comparisons test, where specified. Bar graphs were drawn as mean ± standard deviation (SD). GraphPad Prism 6 for Mac OS X was used for statistical analysis and generation of bar graphs of quantified data.

## Results and Discussion

### Metformin regulates glucose uptake and mTORC1 activity in T cells

**Figure 1:**
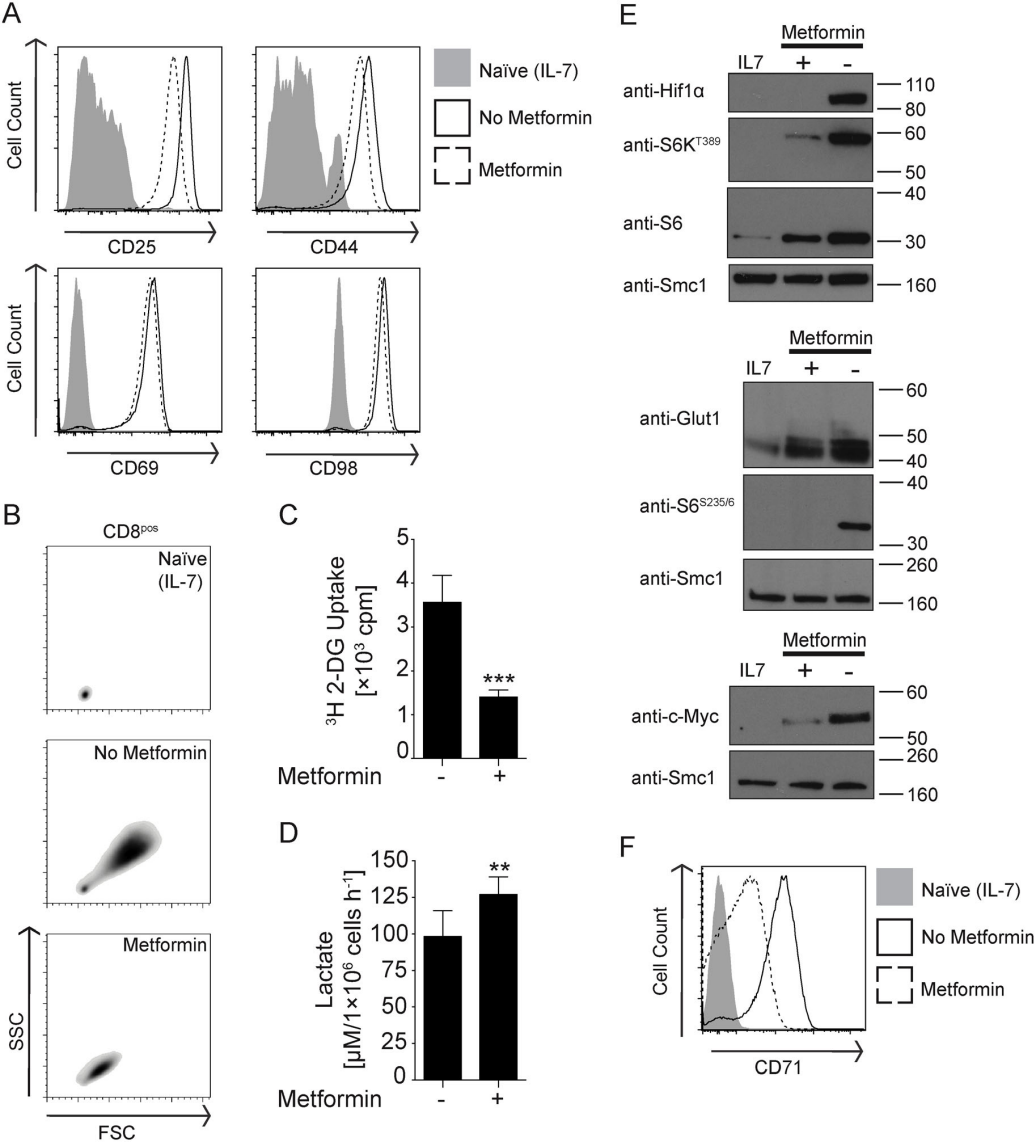
Metformin controls TCR-induced blastogenesis, metabolic reprogramming and mTORC1 signalling in CD8 T cells. Whole lymph node suspensions from OT-I TCR^+^ mice were incubated with IL-7 or activated with ovalbumin-derived SIINFEKL [1 nM] in the absence or presence of 10 mM metformin for 20 h. CD8^pos^ cells were analysed by flow cytometry (**A**) for TCR-induced changes in the surface expression of CD25 (interleukin-2 receptor α chain), CD44 (hyaluronan adhesion receptor), CD69 (early T cell activation) and CD98 (heavy chain of the system L transporter) and (**B**) for changes in cell size using forward and side scatter (FSC and SSC, respectively). (**C, D**) TCR-activated CD8^pos^ T cells were purified by negative selection and then cultured for 3–4 h and subjected to (**C**) radio-labelled glucose ([^3^H] 2-DG) uptake or (**D**) lactic acid production assays. Bar graphs display pooled data from three independent mice in triplicate analysis and show mean ± SD. Statistical analysis was Mann-Whitney test, where **p<0.01 and ***p<0.001. (**E**) Purified CD8^pos^ T cells from IL-7-treated or TCR-activated lymph nodes were rested in conditioned medium (that is medium retained from IL-7-treated or TCR-activated whole lymph node culture) for 3–4 h and then lysed at 1×10^7^ cells mL^−1^ for subsequent immunoblot analysis of indicated phospho- and pan-proteins. Immunoblots are representative of two independent mice. (**F**) CD8^pos^ cells were analysed for the expression of CD71 (transferrin receptor) following TCR activation for 20 h. Bi-parametric plots and histograms are representative of at least three independent experiments.

To understand the effects of metformin on T cells, CD8^+^ OT-I TCR^+^ T cells were activated by the T cell antigen receptor (TCR) ligand SIINFEKL in the absence or presence of 10 mM metformin. T cells respond to antigen receptor triggering by up-regulating expression of the adhesion molecule CD69 and up-regulating expression of CD98, a key subunit of System L amino acid transporters (**Figure 1A**). Antigen activated T cells exposed to metformin also up-regulated expression of CD69 and CD98 and their response was comparable to that of control antigen receptor activated T cells (**Figure 1A**). Metformin treated T cells also respond to TCR engagement by increasing expression of the interleukin-2 receptor (IL-2R) α chain CD25 and the hyaluronan adhesion receptor CD44 (**Figure 1A**). However metformin treatment caused a modest decrease in the expression of these receptors compared to the normal response of antigen activated T cells (**Figure 1A**). Moreover, a striking effect was that metformin-treated T cells did not undergo normal blastogenesis in response to TCR engagement (**Figure 1B**).

T cells respond to antigen by increasing glucose uptake and by making a switch from oxidative phosphorylation to aerobic glycolysis [3], [25], [26]. Metformin-treated antigen receptor activated T cells showed reduced glucose uptake but increased lactate output compared to control antigen-activated cells (**Figure 1C and D**). In T cells, glucose uptake is mediated by the glucose transporter Glut1, whose expression is regulated by the transcription factors c-Myc and hypoxia-inducible factor-1α (HIF-1α) [3], [25]. Metformin treatment blocked TCR-induced expression of c-Myc, HIF-1α and Glut1 (**Figure 1E**). We also examined the impact of metformin on the activity of mTORC1 in TCR-activated T cells. mTORC1 activity was monitored by assessing the phosphorylation of the mTORC1 substrate sequence in p70 S6-Kinase 1 (p70S6K1^T389^) and phosphorylation of the S6K substrate sequence in the S6 ribosomal subunit (S6^S235/6^). **Figure 1E** shows that metformin treatment inhibited TCR-induced phosphorylation of p70S6K^T389^ and S6^S235/6^. Metformin-treated cells also expressed lower levels of S6 protein, suggesting that metformin also decreased TCR-induced ribosomal biogenesis (**Figure 1E**). One other key metabolic change that normally accompanies T cell activation is the induction of expression of the transferrin receptor CD71. **Figure 1F** shows that TCR-induced up-regulation of CD71 was severely reduced in cells treated with metformin compared to control antigen receptor activated cells.

### AMPK-independent actions of metformin in T cells

**Figure 2:**
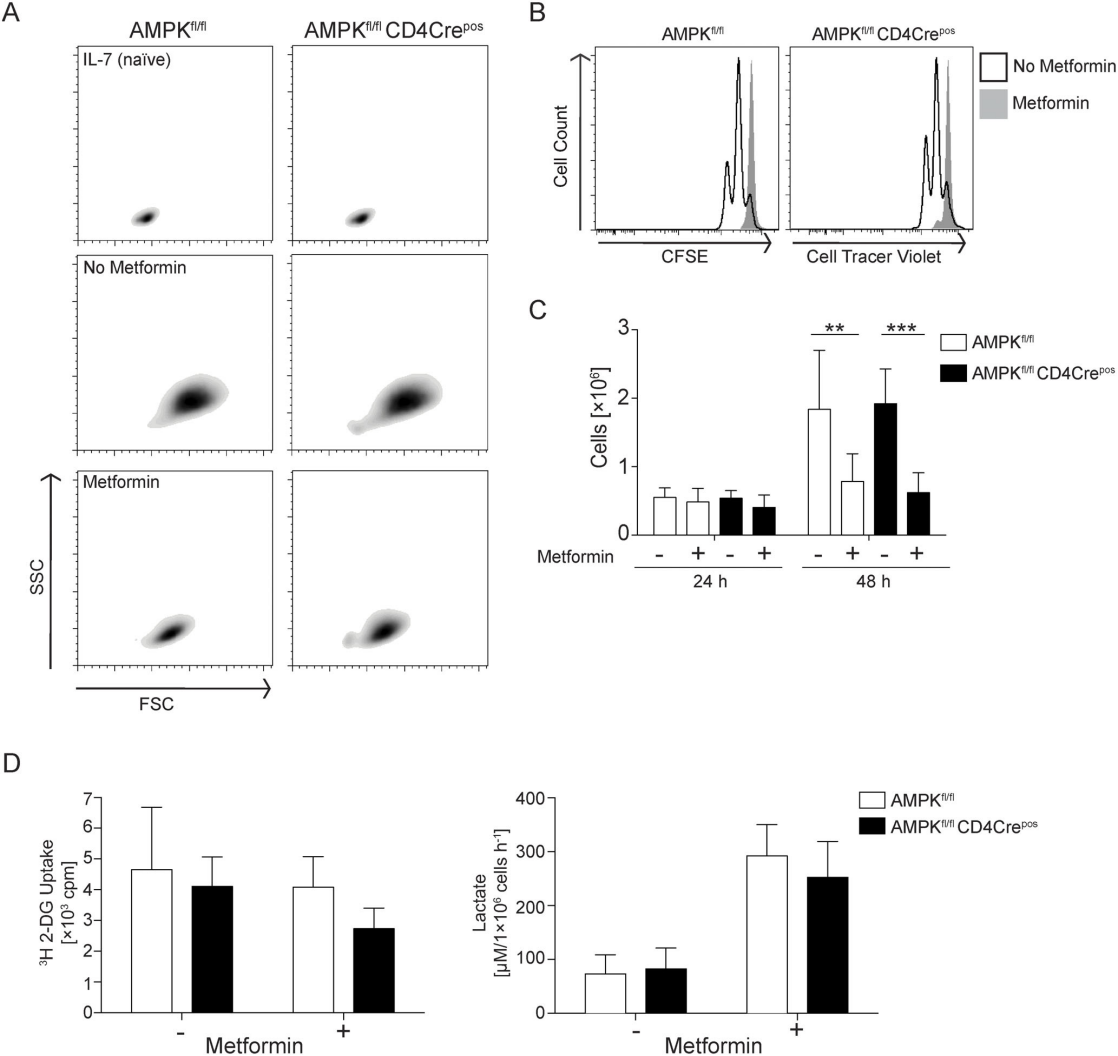
Metformin effects on metabolism and proliferation are independent of AMPK in T cell effectors. (**A**) Cell suspensions of lymph nodes from AMPKα1^fl/fl^ or AMPKα1^fl/fl^ CD4-Cre^+^ mice bearing the OT-I TCR were activated with 1 nM SIINFEKL and treated as described in Figure 1A. CD8^pos^ cells were analysed by flow cytometry for TCR-induced changes in cell size using forward and side scatter (FSC and SSC, respectively). (**B**) Lymph node cells from AMPKα1^fl/fl^ or AMPKα1^fl/fl^ CD4-Cre^pos^ mice bearing OT-I TCR were labelled with CFSE or Cell Tracer Violet, respectively, and mixed in a one-to-one-ratio. Co-cultures were then activated with 1 nM SIINFEKL for up to 72 h in the absence or presence of 10 mM metformin. CD8^pos^ cells were analysed for proliferation-induced dilution of loaded fluorescent dyes using flow cytometry. Data are representative of three independent experiments. (**C**) *In-vitro*-generated AMPKα1^fl/fl^ or AMPKα1^fl/fl^ CD4-Cre^pos^ CTL were treated without or with metformin [10 mM] for 48 h. The starting cell number was 2×10^5^. Proliferation was assessed over the period of treatment by cell counts using flow cytometry. Bar graph displays pooled data of four independent culture experiments. Two-way ANOVA with Bonferroni’s multiple comparisons test was used to determine statistical differences with **p<0.01 and ***p<0.001 between treatments. (**D**) One million IL-2-differentiated AMPKα1^fl/fl^ and AMPKα1^fl/fl^ CD4Cre^pos^ CTL were cultured in absence or presence of metformin [10 mM] for 48 h and subjected to [^3^H] 2-DG uptake or lactic acid production assays. Bar graphs display pooled data of three independent mice analysed in triplicate and are mean ± SD.

The actions of metformin on lymphocytes are interpreted to reflect the impact of this drug on AMPK [13]. To directly examine the role of AMPK as the effector of metformin, we compared the effects of metformin on wild type versus AMPK-null T cells. T cells only express the AMPKα1 catalytic isoform [10], [27]. Accordingly, we could study the role of AMPK as a mediator of metformin actions using isolated AMPKα1 null T cells from mice with a T cell selective deletion of AMPKα1 (CD4Cre^+^ AMPKα1^fl/fl^ (AMPKα1^null^) mice) [10], [28]. The data in **Figure 2** show that metformin blocked T cell blastogenesis and proliferation in both wild type and AMPKα1^null^ naïve TCR-triggered T cells (**Figure 2A and B**). Hence, the ability of metformin to block T cell growth and proliferation is not mediated by AMPK.

In further experiments, we compared the effects of metformin on IL-2 induced growth and proliferation of AMPKα1-sufficient and -deficient cytotoxic T cells (CTL). IL-2 controls effector T cell differentiation and antigen-receptor-activated CD8 T cells cultured in IL-2 clonally expand and differentiate to CTL [29]. The rationale for looking at the effects of metformin on IL-2 signalling in CTL is that these cells have high rates of glucose uptake and are highly glycolytic and it is not clear if they would show any sensitivity to metformin, which inhibits cellular metabolism through the inhibition of respiratory chain complex I. Moreover, IL-2 is a key pro-inflammatory cytokine for effector CTL differentiation *in* *vivo* [29] and hence understanding the impact of metformin on IL-2 responses in T cells might give insights as to why this drug can modify effector/memory T cell differentiation *in* *vivo*. **Figure 2C** addresses this issue and shows that metformin inhibits IL-2-induced clonal expansion of CTL. Moreover, the inhibitory effects of metformin were comparable in AMPKα1-sufficient and -deficient CTL.

**Figure 3:**
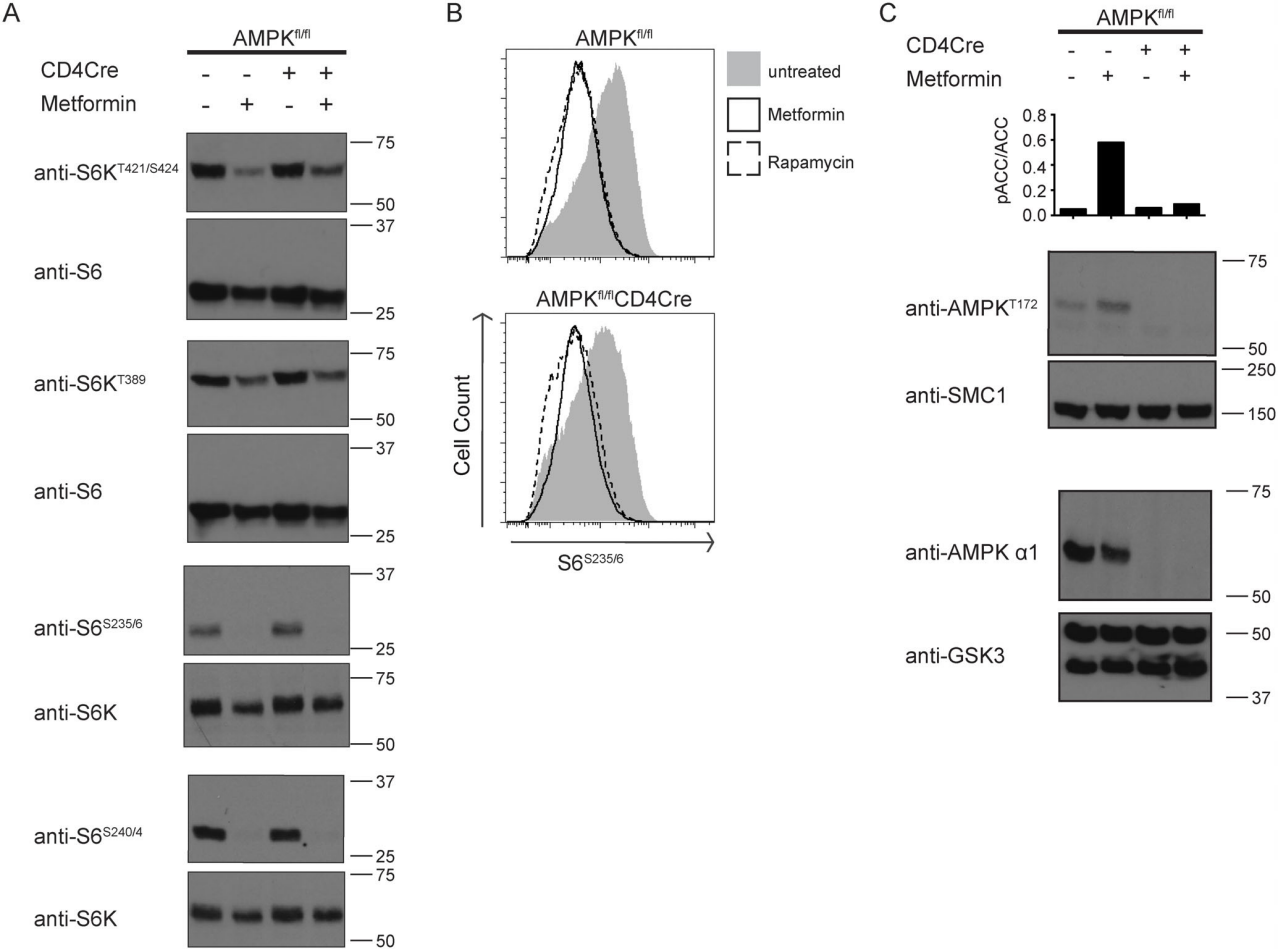
Metformin blocks mTORC1 signalling independent of AMPK in lymphoblasts. CTLs differentiated in IL-2 were treated without or with metformin [10 mM] for 48 h. Protein extracts (1×10^7^ cells mL^−1^) were subject to immunoblot analysis for indicated phospho- and pan-proteins. (**A**) Changes in mTORC1 signalling were assessed by the phosphorylation levels of S6K on T389, T421/S424 and S6 on S235/6 and S240/4. S6K and S6 proteins were used as loading control for equal number of cells between conditions. Molecular weight markers in kD are shown to the right of immunoblot panels. (**B**) Intracellular S6^S235/6^ in CTL left untreated, treated with metformin [10 mM] or rapamycin [20 nM]. Data are representative of at least three independent experiments. (**C**) Activation of AMPK was assessed by induction of phosphorylation of acetyl-CoA carboxylase (pACC/ACC) on S79 and AMPK on T172. Anti-AMPKα1 was used to show successful deletion of AMPK function in T cells. SMC1 and GSK3 were used as total loading control. Molecular weight markers are shown to the right of immunoblot panels. Ratio of pACC/ACC was determined by signal detection using the Licor Odyssey system and quantified by signal integration using ImageJ software. Bar graph and immunoblots shown are representative of at least two independent experiments.

Metformin did not inhibit glucose uptake by CTL but significantly increased lactate output of CTL in a response that was not dependent on the expression of AMPKα1 in CTL (**Figure 2D**). We have shown previously that AMPKα1 can function to terminate mTORC1 activity in CTL under conditions of energy stress [10]. **Figure 3** shows that control and AMPKα1^null^ CTL had high levels of mTORC1 activity as assessed by the levels of IL-2-dependent phosphorylation of p70S6K on T389 and T421/S424, S6^S235/6^ and S6^S240/4^. Strikingly, metformin inhibited mTORC1 signalling equally in AMPKα1-sufficient and -deficient CTL (**Figure 3A**). Further, we have assessed levels of S6^S235/6^ in control and AMPK^null^ CTL upon metformin treatment using flow cytometry providing a means of measuring indirectly metformin effects on mTORC1 signalling and compared this with the treatment of CTL using the mTORC1 inhibitor rapamycin. The data in **Figure 3B** reveal that metformin treatment decreased S6^S235/6^ phosphorylation equally in control and AMPK^null^ CTL and to a comparable effect as rapamycin treatment of CTL. Hence, although metformin could activate AMPK in T cells (**Figure 3C**), AMPKα1 does not mediate the inhibitory actions of metformin on mTORC1 activity in T cells.

## Conclusion

The present study shows that metformin has direct effects on T cells to block their blastogenesis and proliferation. Metformin acts to suppress these T cell responses, because it blocks key metabolic changes triggered by engagement of the T cell antigen receptor complex. Metformin was also able to supress the proliferative response induced by the cytokine IL-2 in effector cytotoxic T cells. Metformin-treated antigen receptor activated T cells have complex metabolic defects and fail to increase glucose uptake or express transferrin receptors. This effect of metformin on T cell metabolism could be explained in part by its effect on mTORC1 activity [3] but could also reflect that metformin-treated T cells cannot respond to T cell activation to normally express the transcription factors c-Myc and HIF-1α. These are key transcription factors that control the expression of nutrient transporters in T cells [3], [25].

It has been shown that metformin treatment can promote memory T cell responses [13] and it was assumed that the immune-modulatory actions of metformin reflected the role of AMPK in memory T cells. Indeed, subsequent studies showed that AMPK is required for the formation of CD8 memory T cells as it restrains mTORC1 activity under conditions of glucose energy stress [10]. Nevertheless, despite the reciprocal effects of metformin treatment and AMPK deletion on memory T cell responses there has been no direct assessment of the role of AMPK as a mediator of metformin action in T cells. The current study addresses this issue and shows that metformin has potent effects as an immunosuppressant independently of AMPK. The present results thus afford the insight that experiments with metformin inform about the importance of metabolic signalling for T cell biology but do not inform about the role of AMPK. How does metformin exert its immune-modulatory actions if not via AMPK? In this context, the direct target for metformin is respiratory chain complex I. It is known that naïve T cells are dependent on oxidative phosphorylation for ATP generation and hence the sensitivity of these cells to metformin would reflect this. It was intriguing that the present data show that effector CTL, cells that are highly glycolytic, were also sensitive to metformin. Thus, although the immune activation of T cells is associated with a switch to glycolysis, the present results argue that metabolic pathways channelled via respiratory chain complex I are important for both naïve and effector T cells.

## References

[1] Pearce EL, Poffenberger MC, Chang CH, Jones RG (2013) Fueling Immunity: Insights into Metabolism and Lymphocyte Function. Science 342: 1242454–1242454 10.1126/science.1242454 24115444 PMC4486656

[2] MacIver NJ, Michalek RD, Rathmell JC (2013) Metabolic Regulation of T Lymphocytes. Annu Rev Immunol 31: 259–283 10.1146/annurev-immunol-032712-095956 23298210 PMC3606674

[3] Finlay DK, Rosenzweig E, Sinclair LV, Feijoo-Carnero C, Hukelmann JL, et al. (2012) PDK1 regulation of mTOR and hypoxia-inducible factor 1 integrate metabolism and migration of CD8+ T cells. Journal of Experimental Medicine. doi:10.1084/jem.20112607. 10.1084/jem.20112607 PMC3526360 23183047

[4] Macintyre AN, Finlay D, Preston G, Sinclair LV, Waugh CM, et al (2011) Protein Kinase B Controls Transcriptional Programs that Direct Cytotoxic T Cell Fate but Is Dispensable for T Cell Metabolism. Immunity 34: 224–236 10.1016/j.immuni.2011.01.012 21295499 PMC3052433

[5] Powell JD, Delgoffe GM (2010) The Mammalian Target of Rapamycin: Linking T Cell Differentiation, Function, and Metabolism. Immunity 33: 301–311 10.1016/j.immuni.2010.09.002 20870173 PMC2962404

[6] Sinclair LV, Finlay D, Feijoo C, Cornish GH, Gray A, et al (2008) Phosphatidylinositol-3-OH kinase and nutrient-sensing mTOR pathways control T lymphocyte trafficking. Nature Immunology 9: 513–521 10.1038/ni.1603 18391955 PMC2857321

[7] Araki K, Turner AP, Shaffer VO, Gangappa S, Keller SA, et al (2009) mTOR regulates memory CD8 T-cell differentiation. Nature 460: 108–112 10.1038/nature08155 19543266 PMC2710807

[8] He S, Kato K, Jiang J, Wahl DR, Mineishi S, et al (2011) Characterization of the Metabolic Phenotype of Rapamycin-Treated CD8+ T Cells with Augmented Ability to Generate Long-Lasting Memory Cells. PLoS ONE 6: e20107 10.1371/journal.pone.0020107.g008 21611151 PMC3096660

[9] MacIver NJ, Blagih J, Saucillo DC, Tonelli L, Griss T, et al (2011) The Liver Kinase B1 Is a Central Regulator of T Cell Development, Activation, and Metabolism. The Journal of Immunology 187: 4187–4198 10.4049/jimmunol.1100367 21930968 PMC3206094

[10] Rolf J, Zarrouk M, Finlay DK, Foretz M, Viollet B, et al. (2013) AMPKα1: A glucose sensor that controls CD8 T-cell memory. Eur J Immunol: n/a–n/a. doi:10.1002/eji.201243008. 10.1002/eji.201243008 PMC3734624 23310952

[11] Hardie DG, Ross FA, Hawley SA (2012) AMPK: a nutrient and energy sensor that maintains energy homeostasis. Nature Publishing Group 13: 251–262 10.1038/nrm3311 PMC5726489 22436748

[12] Kang KY, Kim Y-K, Yi H, Kim J, Jung H-R, et al. (2013) Metformin downregulates Th17 cells differentiation and attenuates murine autoimmune arthritis. International Immunopharmacology: 1–8. doi:10.1016/j.intimp.2013.03.020. 10.1016/j.intimp.2013.03.020 23557965

[13] Pearce EL, Walsh MC, Cejas PJ, Harms GM, Shen H, et al (2009) Enhancing CD8 T-cell memory by modulating fatty acid metabolism. Nature 460: 103–107 10.1038/nature08097 19494812 PMC2803086

[14] Michalek RD, Gerriets VA, Jacobs SR, Macintyre AN, MacIver NJ, et al (2011) Cutting Edge: Distinct Glycolytic and Lipid Oxidative Metabolic Programs Are Essential for Effector and Regulatory CD4+ T Cell Subsets. The Journal of Immunology 186: 3299–3303 10.4049/jimmunol.1003613 21317389 PMC3198034

[15] Nath N, Khan M, Paintlia MK, Hoda MN, Giri S (2009) Metformin Attenuated the Autoimmune Disease of the Central Nervous System in Animal Models of Multiple Sclerosis. The Journal of Immunology 182: 8005–8014 10.4049/jimmunol.0803563 19494326 PMC2965405

[16] Green AS, Chapuis N, Trovati Maciel T, Willems L, Lambert M, et al (2010) The LKB1/AMPK signaling pathway has tumor suppressor activity in acute myeloid leukemia through the repression of mTOR-dependent oncogenic mRNA translation. Blood 116: 4262–4273 10.1182/blood-2010-02-269837 20668229

[17] Rosilio CL, Lounnas N, Nebout M, Imbert V, Hagenbeek T, et al. (2013) The metabolic perturbators metformin, phenformin and AICAR interfere with the growth and survival of murine PTEN-deficient T cell lymphomas and human T-ALL/T-LL cancer cells. CANCER LETTERS: 1–13. doi:10.1016/j.canlet.2013.04.015. 10.1016/j.canlet.2013.04.015 23612073

[18] Grimaldi C, Chiarini F, Tabellini G, Ricci F, Tazzari PL, et al (2011) AMP-dependent kinase/ mammalian target of rapamycin complex 1 signaling in T-cell acute lymphoblastic leukemia: therapeutic implications. 26: 91–100 10.1038/leu.2011.269 21968881

[19] Owen MR, Doran E, Halestrap AP (2000) Evidence that metformin exerts its anti-diabetic effects through inhibition of complex 1 of the mitochondrial respiratory chain. Biochem J 348 Pt 3: 607–614. PMC1221104 10839993

[20] Foretz M, Hébrard S, Leclerc J, Zarrinpashneh E, Soty M, et al (2010) Metformin inhibits hepatic gluconeogenesis in mice independently of the LKB1/AMPK pathway via a decrease in hepatic energy state. J Clin Invest 120: 2355–2369 10.1172/JCI40671DS1 20577053 PMC2898585

[21] Ben Sahra I, Regazzetti C, Robert G, Laurent K, Le Marchand-Brustel Y, et al (2011) Metformin, Independent of AMPK, Induces mTOR Inhibition and Cell-Cycle Arrest through REDD1. Cancer Research 71: 4366–4372 10.1158/0008-5472.CAN-10-1769 21540236

[22] Miller RA, Chu Q, Xie J, Foretz M, Viollet B, et al (2013) Biguanides suppress hepatic glucagon signalling by decreasing production of cyclic AMP. Nature 494: 256–260 10.1038/nature11808 23292513 PMC3573218

[23] Sinclair LV, Rolf J, Emslie E, Shi Y-B, Taylor PM, et al. (2013) Control of amino-acid transport by antigen receptors coordinates the metabolic reprogramming essential for T cell differentiation. Nature Immunology: 1–11. doi:10.1038/ni.2556. 10.1038/ni.2556 PMC3672957 23525088

[24] Holman GD, Kozka IJ, Clark AE, Flower CJ, Saltis J, et al (1990) Cell surface labeling of glucose transporter isoform GLUT4 by bis-mannose photolabel. Correlation with stimulation of glucose transport in rat adipose cells by insulin and phorbol ester. J Biol Chem 265: 18172–18179. 2211693

[25] Wang R, Dillon CP, Shi LZ, Milasta S, Carter R, et al (2011) The Transcription Factor Myc Controls Metabolic Reprogramming upon T Lymphocyte Activation. Immunity 35: 871–882 10.1016/j.immuni.2011.09.021 22195744 PMC3248798

[26] Wang R, Green DR (2012) Metabolic checkpoints in activated T cells. Nature Immunology 13: 907–915 10.1038/ni.2386 22990888

[27] Tamas P, Hawley SA, Clarke RG, Mustard KJ, Green K, et al (2006) Regulation of the energy sensor AMP-activated protein kinase by antigen receptor and Ca2+ in T lymphocytes. J Exp Med 203: 1665–1670 10.1084/jem.20052469 16818670 PMC2118355

[28] Zarrouk M, Rolf J, Cantrell DA (2013) LKB1 Mediates the Development of Conventional and Innate T Cells via AMP-Dependent Kinase Autonomous Pathways. PLoS ONE 8: e60217 10.1371/journal.pone.0060217.g006 23533675 PMC3606301

[29] Zhang N, Bevan MJ (2011) CD8+ T Cells: Foot Soldiers of the Immune System. Immunity 35: 161–168 10.1016/j.immuni.2011.07.010 21867926 PMC3303224

